# The Intersection of Autoimmunity and Neurology: Insights From a Case of Striatal Lupus Encephalitis

**DOI:** 10.7759/cureus.68743

**Published:** 2024-09-05

**Authors:** Tze Hui Soo, Subapriya Suppiah, Muhammad Furhan Zamri

**Affiliations:** 1 Department of Radiology, Faculty of Medicine and Health Sciences, Universiti Putra Malaysia, Serdang, MYS

**Keywords:** limbic encephalitis, neuropsychiatric systemic lupus erythematosus (npsle), nmdar encephalitis, striatal lupus encephalitis, systemic lupus erythematosus

## Abstract

Systemic lupus erythematosus (SLE) is a multifactorial autoimmune disorder predominantly affecting women, characterized by the production of autoantibodies against various nuclear antigens, leading to widespread immune dysregulation and multisystem involvement. Among its complex manifestations, neuropsychiatric systemic lupus erythematosus (NPSLE) represents a particularly challenging aspect of the disease due to its wide spectrum of neurological and psychiatric symptoms. This case report presents a rare instance of striatal lupus encephalitis, a severe subtype of NPSLE, in a 32-year-old woman, highlighting its distinct clinical and radiological features. The patient initially developed bilateral ocular occlusive vasculitis and later presented with acute right-sided hemiparesis and facial asymmetry. Magnetic resonance imaging (MRI) revealed bilateral symmetrical T2-weighted and fluid-attenuated inversion recovery (FLAIR) hyperintense signals in the basal ganglia, consistent with striatal lupus encephalitis. No white matter hyperintensity or vasculitis changes were seen. Cerebrospinal fluid analysis revealed markedly elevated protein levels, though no infectious organism was identified. The patient was treated with high-dose prednisolone, alongside empirical antibiotic and antiviral therapy to address potential meningoencephalitis. Remarkably, she made a full recovery from her stroke-like symptoms. Despite its rarity, the identification of striatal lupus encephalitis is critical due to the severe and potentially irreversible nature of the neurological damage. This case underscores the importance of a comprehensive diagnostic approach, integrating clinical, serological, and neuroimaging findings to differentiate striatal lupus encephalitis from other neuropsychiatric conditions associated with SLE. Its management typically involves aggressive immunosuppressive therapy, with intravenous methylprednisolone being the first-line treatment. The case also illustrates the potential for recovery with prompt and appropriate treatment, as evidenced by the complete resolution of neurological symptoms and MRI findings at follow-up.

## Introduction

Systemic lupus erythematosus (SLE) is a multifactorial autoimmune disorder predominantly affecting women of childbearing age, with an estimated female-to-male ratio of approximately 9:1, particularly those of African, Asian, and Hispanic descent. This gender disparity is believed to be influenced by hormonal factors, particularly oestrogen, which modulates immune responses, potentially contributing to the heightened susceptibility in women [1]. SLE is characterized by the production of autoantibodies against a variety of nuclear antigens. This autoimmune response is triggered by a combination of genetic predisposition, hormonal influences, and environmental factors such as infections and ultraviolet radiation. Laboratory tests play a crucial role in the diagnosis, with anti-nuclear antibodies (ANA) being positive in over 95% of the cases. Anti- double-stranded DNA (dsDNA) and anti-Smith (anti-Sm) antibodies are highly specific for SLE and are often associated with disease activity, particularly renal involvement. These autoantibodies lead to the formation of immune complexes that deposit in various tissues, causing inflammation and subsequent tissue damage. Complement levels, particularly C3 and C4, are typically low during active disease phases and can serve as markers for disease monitoring. SLE involves widespread immune dysregulation, including the activation of autoreactive B cells and T cells, impaired clearance of apoptotic cells, and the overproduction of pro-inflammatory cytokines, which contribute to the systemic nature of the disease [2]. Clinically, SLE presents with a wide array of symptoms, ranging from constitutional manifestations such as fever, fatigue, and weight loss, to more specific organ involvement. The disease can affect virtually any organ, with common manifestations including malar rash, arthritis, nephritis, and neurological symptoms such as seizures or psychosis. Additionally, haematological abnormalities like anaemia, leukopenia, and thrombocytopenia are frequently observed [3]. Cutaneous involvement, one of the most prevalent manifestations of SLE, often presents as malar rash, discoid lesions, or photosensitivity. The histopathological examination of skin biopsies is crucial for confirming cutaneous lupus erythematosus, providing insight into disease activity, and guiding treatment decisions. Although rare, lupus enteritis, characterized by abdominal pain, nausea, and vomiting, can occur and may be the sole active manifestation of SLE, highlighting the diverse and unpredictable nature of the disease [4,5]. Diagnosing SLE is challenging due to its heterogeneous clinical presentations, which can affect virtually any organ system. The American College of Rheumatology (ACR) and the Systemic Lupus International Collaborating Clinics (SLICC) have established classification criteria that include a combination of clinical and immunological parameters.

## Case presentation

A 32-year-old woman, recently diagnosed with SLE one month ago, was referred to a tertiary care centre for continued management. Laboratory investigations revealed positive ANA and anti-dsDNA with concomitantly low levels of C3 and C4. The extractable nuclear antigen panel, however, was within normal limits. Clinically, she initially presented with a two-month history of persistent oral ulcers, arthralgia predominantly affecting the knees and small joints of the hands, and associated morning stiffness. Additionally, she reported significant hair loss and photosensitivity, though she denied any chest pain, seizures, dysphagia, or movement disorders. There was no notable family history of malignancy or connective tissue disease. Physical examination revealed a malar rash across both cheeks, as well as a discoid rash on the dorsum of the right hand and bilateral lower extremities. The remainder of the systemic and neurological examinations were unremarkable. She was treated with hydroxychloroquine and prednisolone, which led to a resolution of her joint pain and cutaneous manifestations. Subsequently, she developed right-sided blurred vision accompanied by a right temporal headache and fever. Notably, there were no associated symptoms such as diplopia, photophobia, or neck stiffness. Fundoscopy examination revealed cotton wool spots and vasculitis in all quadrants of the right eye and the superonasal quadrant of the left eye. Both optic discs appeared pink with a normal cup-to-disc ratio. Given these findings, a diagnosis of bilateral ocular occlusive vasculitis secondary to systemic lupus erythematosus (SLE) retinopathy and right eye macular oedema, likely optic neuritis, was made. Raised inflammatory markers, including erythrocyte sedimentation rate and C-reactive protein, supported the inflammatory nature of her condition. A contrast-enhanced computed tomography (CT) scan of the brain and orbits showed no evidence of abnormal leptomeningeal enhancement or space-occupying lesion. She was subsequently treated with intravenous methylprednisolone (MTP) for three days, according to the local protocol, which resulted in significant improvement in her ocular symptoms, leading to her discharge.

**Figure 1:**
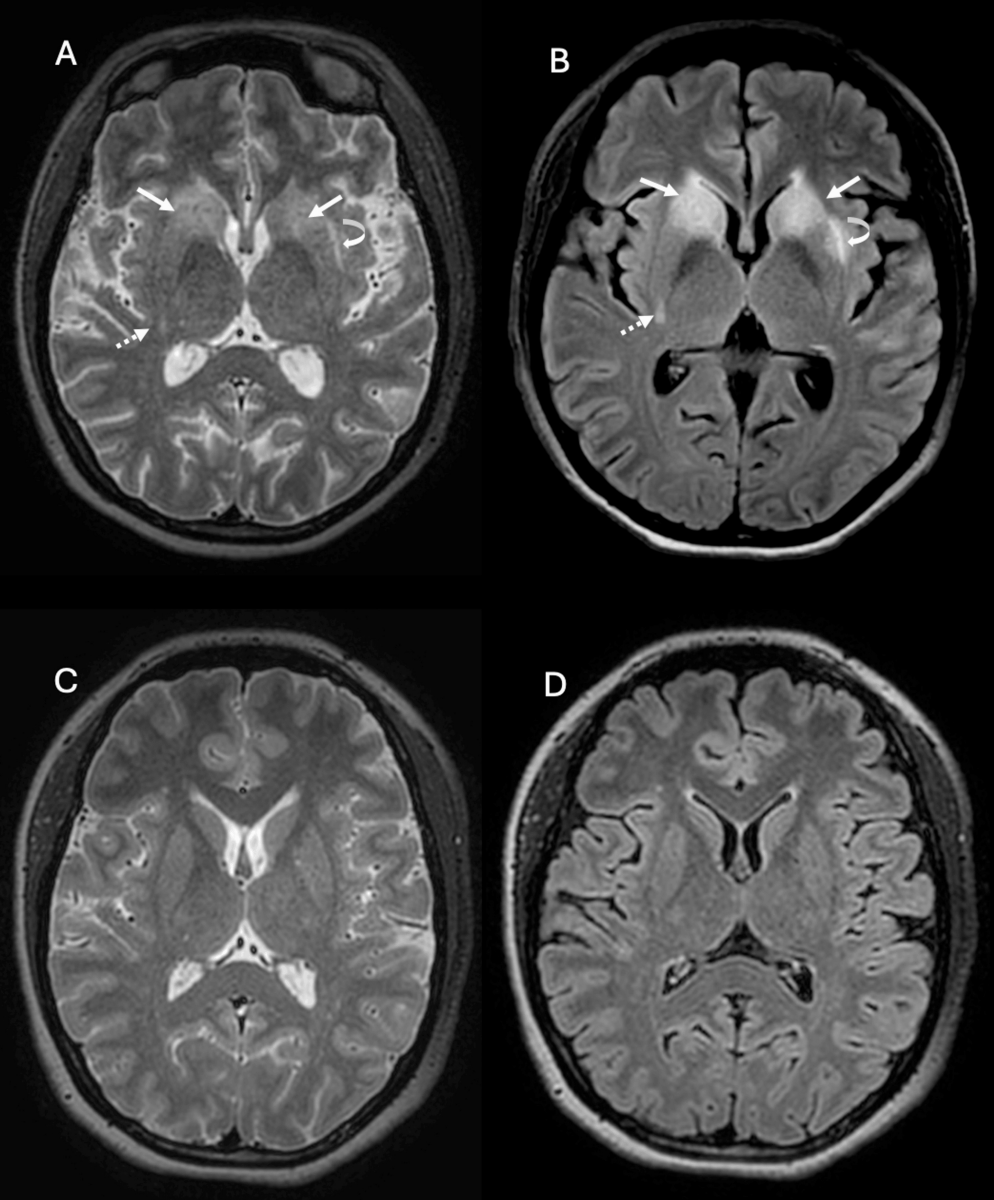
Axial MRI brain images in T2-weighted (A) and fluid-attenuated inversion recovery (FLAIR) (B) sequences showing hyperintense signals in both basal ganglia (arrow), left external capsule (curved arrow), and posterior right putamen (dashed arrow). Mild effacement of the frontal horn of both lateral ventricles is also noted. A follow-up study (C, D) reveals complete resolution of the hyperintense signals, indicating a favourable response to prompt and appropriate treatment.

However, within a few hours post-discharge, she presented to the emergency department with an acute onset of right-sided hemiparesis, facial asymmetry, and slurred speech, persisting for one hour prior to arrival. Despite these symptoms, she remained alert and conscious. An urgent magnetic resonance imaging (MRI) of the brain was performed to exclude an acute stroke. The MRI revealed symmetrical T2-weighted and fluid-attenuated inversion recovery (FLAIR) hyperintense signals in bilateral caudate head, bilateral anterior limb of internal capsule, bilateral anterior putamen, left external capsule, and posterior right putamen (Figure 1A, 1B), with no avid enhancement on post-contrast sequences. Mild effacement of the frontal horn of both lateral ventricles was noted.

**Figure 2:**
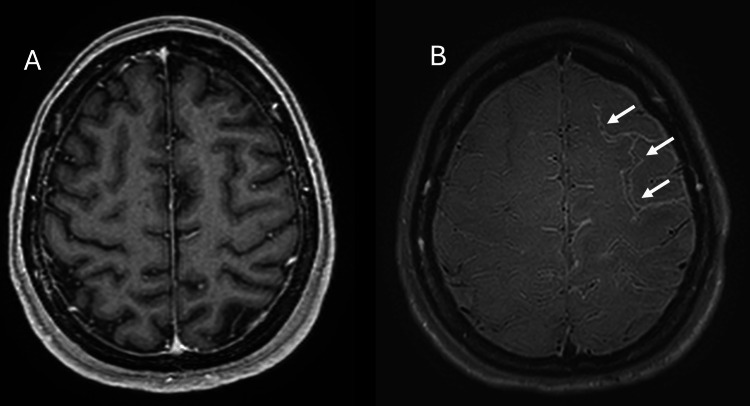
Axial MRI brain images in T1-weighted post-gadolinium (A) and vessel wall black-blood imaging (2B) sequences. Patchy leptomeningeal enhancement is observed in the left fronto-parietal region (arrow in B), with the vessel wall black-blood imaging sequence providing the best visualization.

Importantly, there were no blooming artefacts suggestive of haemorrhage. Additionally, there was patchy leptomeningeal enhancement in the left fronto-parietal region, best visualized on vessel wall black-blood imaging sequences (Figure 2A, 2B).

No irregularities or beaded appearances were observed in the cerebral or carotid vessels, and there was no evidence of vessel occlusion within the circle of Willis or thrombosis in the superficial and deep cerebral venous systems. At this juncture, a diagnosis of striatal lupus encephalitis was made, and empirical treatment with antibiotic (ceftriaxone) and antiviral (acyclovir) medication was initiated to cover for possible concomitant meningoencephalitis in addition to her prednisolone, which was planned at 60mg daily for one week, followed by a tapering dose of 40mg daily for two weeks. A lumbar puncture was performed subsequently, revealing a normal opening pressure of 19cm H_2_O but a markedly elevated cerebrospinal fluid (CSF) protein level of 2776 mg/L (normal range: 150-400 mg/L). The cerebrospinal fluid (CSF) analysis for acid-fast bacillus, *Mycobacterium* culture and sensitivity (C&S) testing, viral panels (including India ink, Venereal Disease Research Laboratory, and *Cryptococcus*), and cytology were all negative. White blood cell and lymphocyte count were negative. The patient had a smooth recovery with complete resolution of her stroke-like symptoms and was discharged after a one-week hospital stay. A follow-up MRI at six months showed complete resolution of the basal ganglia signal changes (Figure 1C, 1D). She has remained well and compliant with her medications, including hydroxychloroquine, azathioprine, and prednisolone.

## Discussion

Cerebral lupus, or neuropsychiatric systemic lupus erythematosus (NPSLE), is a significant and complex manifestation of SLE, encompassing a wide spectrum of neurological and psychiatric symptoms. NPSLE indicates a notably high prevalence among SLE patients, with reports suggesting that between 40% and 90% of individuals may experience neuropsychiatric symptoms at some stage of their disease [6,7]. The clinical presentation of NPSLE is highly variable, including symptoms such as cognitive dysfunction, mood disorders, anxiety, headaches, and more severe manifestations like seizures, psychosis, and cerebrovascular events [8]. The pathophysiology of NPSLE involves complex autoimmune processes where autoantibodies, particularly anti-NR2A/B, target neuronal components, leading to neuroinflammation and subsequent neuronal damage [9]. Additionally, the disruption of the blood-brain barrier by inflammatory cytokines and immune complexes exacerbates the neurological impairment seen in these patients. The CSF fluid of these patients often reveals elevated inflammatory markers, suggesting systemic inflammation as a contributory factor to central nervous system involvement.

Striatal lupus encephalitis is a rare but severe subtype of NPSLE, specifically characterized by inflammation of the striatum, leading to a distinctive set of neurological and psychiatric symptoms. This condition is exceedingly uncommon, with only a few cases documented in the literature, making precise epidemiological data scarce [10,11]. The hallmark of striatal lupus encephalitis on MRI is similar to those observed in our index case [11]. The pathophysiology of striatal lupus encephalitis involves complex autoimmune processes, where autoantibodies, such as those against N-methyl-D-aspartate receptor (NMDAR), play a pivotal role. These autoantibodies are thought to disrupt synaptic function and promote neuroinflammation within the striatum, leading to the observed clinical manifestations [12]. Notably, this condition shares pathophysiological similarities with autoimmune encephalitis, particularly NMDAR encephalitis, which is also characterized by psychiatric disturbances and striatal involvement. Diagnostically, the differentiation of striatal lupus encephalitis from other forms of NPSLE is primarily reliant on the distinct neuroimaging patterns and the presence of these specific autoantibodies [10]. Unlike more generalized forms of cerebral lupus, striatal lupus encephalitis directly impacts motor control and behaviour due to its localized effect on the basal ganglia, which governs these functions.

The differential diagnosis of striatal lupus encephalitis includes a variety of neuropsychiatric conditions, particularly those associated with SLE and other autoimmune encephalitides. Conditions such as neuropsychiatric lupus cerebritis, limbic encephalitis, and NMDAR encephalitis must be considered. However, striatal lupus encephalitis can be distinguished by its unique radiological signature. On MRI, this condition is characterized by bilateral symmetric hyperintensities in the caudate nucleus and putamen on FLAIR and T2-weighted sequences, which are not typically seen in other forms of lupus-related neuropsychiatric conditions [11]. In contrast, lupus cerebritis often presents with more diffuse white matter hyperintensities, while limbic encephalitis primarily involves the medial temporal lobes [13,14]. Additionally, NMDAR encephalitis may show more widespread cortical and subcortical abnormalities, without the specific striatal involvement that characterizes striatal lupus encephalitis [12]. The presence of autoantibodies, such as those targeting NMDAR, further complicates the differential diagnosis, as these can be found in both striatal lupus encephalitis and other autoimmune encephalitides. Therefore, a comprehensive evaluation combining clinical, serological, and neuroimaging findings is essential to accurately differentiate striatal lupus encephalitis from other neuropsychiatric conditions associated with SLE [10].

The management of SLE, particularly when complicated by neuropsychiatric manifestations, requires a tailored and multidisciplinary approach. Standard treatment regimens for SLE typically include immunosuppressive therapies, such as corticosteroids, cyclophosphamide, and mycophenolate mofetil, aimed at controlling systemic inflammation and preventing disease progression [15]. In cases of NPSLE, including striatal lupus encephalitis, more aggressive immunosuppressive strategies are often employed due to the severe and potentially irreversible nature of the neurological damage. Intravenous methylprednisolone is frequently used as first-line therapy, often followed by plasmapheresis or intravenous immunoglobulins (IVIG) in refractory cases [10]. The use of rituximab, a monoclonal antibody targeting B cells, has also been explored in severe cases of NPSLE and striatal lupus encephalitis with promising results [11]. Additionally, addressing specific symptoms such as seizures or psychiatric disturbances requires adjunctive treatments, including antiepileptic drugs and antipsychotics, although these are generally considered supportive rather than curative. Early diagnosis and prompt initiation of therapy are crucial to prevent permanent neurological deficits. The integration of neuroimaging and serological monitoring is essential in guiding treatment decisions and assessing response to therapy. As our understanding of the pathophysiology of striatal lupus encephalitis continues to evolve, there is potential for more targeted therapies that could improve outcomes for patients with this rare but severe manifestation of SLE [16].

## Conclusions

Striatal lupus encephalitis represents a rare and challenging manifestation of SLE characterized by specific neurological symptoms related to the striatum, a critical brain structure involved in motor control and cognition. This condition underscores the complex interplay between autoimmune processes and neuropsychiatric manifestations in SLE. Diagnosis relies on a combination of clinical presentation, imaging studies, and autoimmune markers, while management typically involves a multidisciplinary approach to address both the underlying lupus and the neurological symptoms. Advances in understanding the pathophysiology of striatal lupus encephalitis and early intervention strategies are crucial for improving patient outcomes. Ongoing research is essential to better elucidate the mechanisms behind this condition and to develop more effective therapeutic strategies tailored to the unique needs of affected individuals.
